# Epicardial Fat Expansion in Diabetic and Obese Patients With Heart Failure and Preserved Ejection Fraction—A Specific HFpEF Phenotype

**DOI:** 10.3389/fcvm.2021.720690

**Published:** 2021-09-17

**Authors:** Ahmed Elsanhoury, Vivian Nelki, Sebastian Kelle, Sophie Van Linthout, Carsten Tschöpe

**Affiliations:** ^1^Berlin Institute of Health at Charite (BIH), Universitätsmedizin Berlin, BIH Center for Regenerative Therapies (BCRT), Berlin, Germany; ^2^German Center for Cardiovascular Research (DZHK), Partner Site Berlin, Berlin, Germany; ^3^Department of Cardiology, Campus Virchow Klinikum (CVK), Charité Universitätsmedizin Berlin, Berlin, Germany; ^4^Department of Internal Medicine/Cardiology, German Heart Center Berlin, Berlin, Germany

**Keywords:** heart failure with a preserved ejection fraction, epicardiac adipose tissue, diabetes, obesity, SGLT2, inhibitor, GLP-1 agonists

## Abstract

Heart failure with preserved ejection fraction (HFpEF) is a heterogeneous syndrome with diverse etiologies and pathophysiological factors. Obesity and type 2 diabetes mellitus (T2DM), conditions that coexist frequently, induce a cluster of metabolic and non-metabolic signaling derangements which are in favor to induce inflammation, fibrosis, myocyte stiffness, all hallmarks of HFpEF. In contrast to other HFpEF risk factors, obesity and T2DM are often associated with the generation of enlarged epicardial adipose tissue (EAT). EAT acts as an endocrine tissue that may exacerbate myocardial inflammation and fibrosis *via* various paracrine and vasocrine signals. In addition, an abnormally large EAT poses mechanical stress on the heart *via* pericardial restrain. HFpEF patients with enlarged EAT may belong to a unique phenotype that can benefit from specific EAT-targeted interventions, including life-style modifications and pharmacologically *via* statins and fat modifying anti-diabetics drugs; like metformin, sodium-glucose cotransporter 2 inhibitors, or glucagon-like peptide-1 receptor agonists, respectively.

## Graphical Abstract

Epicardial adipose tissue (EAT)-related heart failure with preserved ejection fraction (HFpEF). Obesity and type 2 diabetes mellitus (T2DM) are common triggers of HFpEF, frequently associated with EAT expansion. EAT plays metabolic and mechanical roles in HFpEF development *via* para/vasocrine factors and pericardial restrain, respectively. Life-style modifications including healthy diet and regular exercise can quash the EAT expansion. Statins, proprotein convertase subtilisin/kexin type 9 (PCSK9) inhibitors and fat-modulating antidiabetics including metformin, sodium-glucose cotransporter 2 (SGLT2) inhibitors and glucagon-like peptide-1 (GLP-1) agonists can target EAT. FFA, free fatty acids; AGEs, advanced glycation end-products; NO, nitric oxide; ROS, reactive oxygen species; Ang-II, angiotensin II; TGF-β, Transforming growth factor beta; MCP-1, monocyte chemoattractant protein 1; IL-6, interleukin 6; TNF-α, tumor necrosis factor alpha. Figure created *via* Servier Medical Art and BioRender tools. 
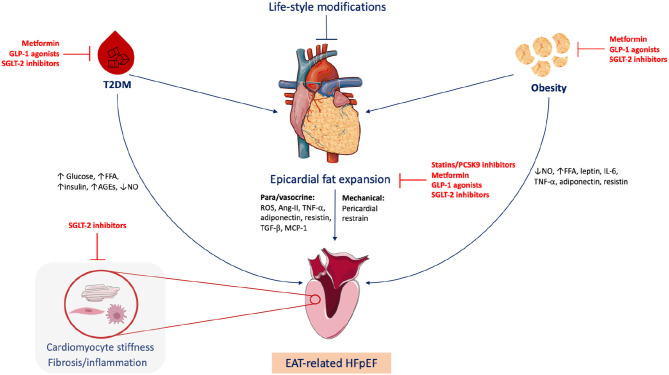

## Introduction

Nearly one-half of heart failure (HF) patients have a preserved ejection fraction (HFpEF), with rising prevalence in the United States of America and Western populations (1, 2). Common hemodynamic features of HFpEF include diastolic dysfunction and reduced ventricular compliance (3). The pathophysiology of HFpEF is complex, exacerbated by a variety of comorbidities including age, hypertension, renal dysfunction, diabetes mellitus (DM), and obesity (4, 5), and may reflect also different phenotypes and differences in pathology (6). This could be especially important for the obese and DM subgroup of HFpEF. In dispersion through large outcome studies and registries, around 80% of HFpEF patients are obese and 20–45% have type 2 DM (T2DM) (7–10). Thirty-percent of HFpEF patients have both obesity and T2DM (11). As such, HFpEF is perceived as an inflammatory cardiometabolic disease, which includes all major mechanisms discussed in HFpEF (12, 13). However, even in this subgroup of HFpEF some patients, differ with respect to the existences of epicardial adipose tissue (EAT). EAT, which is the visceral fat depot of the heart, may play an important extra role in the development of HFpEF (10, 14). Compared to non-obese HFpEF patients, HFpEF patients with obese phenotype show 20–50% higher EAT thickness despite similar body mass index (BMI) (15). The volume of EAT is directly proportional with the presence of atrial fibrillation and T2DM and with myocardial injury biomarkers (16). It has been recognized as a metabolically active depot that affects the myocardium *via* production of cytokines and adipokines (17).

There is growing evidence that HFpEF with enlarged EAT is a clinically relevant HF phenotype that may require specific treatments (15, 17). We hypothesize that EAT quantification would allow the differentiation of HFpEF patients with enlarged EAT. This specific subgroup of patients might benefit from EAT-modifying therapies.

## The Obese HFpEF Phenotype

HFpEF is a systemic disorder involving multiple organ systems where circulating proinflammatory mediators originating from multiple comorbidities trigger abnormalities in both the heart and the skeletal muscles (18). An expansion in body fat mass causes hemodynamic, metabolic, inflammatory, and hormonal disruption, which affect the vascular endothelium and the heart (19, 20). Obesity is a principal HFpEF component *via* triggering a systemic proinflammatory environment and inducing endothelial production of reactive oxygen species and reduces nitric oxide (NO) bioavailability, which especially affect the coronary microvasculature and the neighboring cardiomyocytes (21–23). Moreover, cardiomyocytes have no lipid storage capacity, their exposure to excess blood lipids, typically occurring in obese patients can lead to cardiomyocyte steatosis and reduction of function (14, 24).

Accordingly, HFpEF patients can be further classified into non-obese and obese phenotypes. The latter is characterized by high body mass index >30 kg/m^2^, lower natriuretic peptide levels and higher left ventricular (LV) mass to volume ratio compared to the non-obese phenotype which is more common in the elderly (15). Moreover, obese HFpEF patients have abnormal cardiac and skeletal muscle composition with infiltration of adipose tissue (25). Furthermore, in comparison with non-obese HFpEF patients, obese HFpEF patients have unique pathophysiological features including larger volume overload, abnormal right ventricular-pulmonary arterial coupling, worse exercise capacity, subtle hemodynamic perturbations, increased epicardial fat mass and higher LV filling pressure and exaggerated biventricular remodeling (15, 26, 27). Increased biventricular pressure can be attributed to greater dependence on plasma volume expansion, and enhanced ventricular interaction, and is further amplified as pulmonary pressure load increases (15, 26). This increase in the pulmonary pressure is a consequence of impaired pulmonary vasodilatation that could be related to the existence of adipokines which reduce NO bioavailability (28, 29). However, obese patients can also develop a significant enlargement in EAT, which may represent an additional factor in the HFpEF hemodynamic features due to an increased pericardial restraint.

## Diabetic Cardiomyopathy With HFpEF Phenotype

Like obesity, T2DM plays a fundamental role in the pathophysiology of HFpEF *via* diabetic specific mechanisms which culminate in matrix changes, vascular endothelial dysfunction, and myocardial stiffness, respectively (30, 31). In HFpEF patients, T2DM is associated with poor prognosis manifested in an increased risk of hospitalization for worsening HF and HF-related death (32, 33). In obese T2DM patients, metabolic disturbances including hyperglycemia, lipotoxicity, abundance of advanced glycation end-products (AGEs), and hyperinsulinemia provoke coronary microvascular dysfunction, and the development of HFpEF (21, 30). Hyperglycemia impairs endothelial NO generation and reduces cyclic guanosine monophosphate (cGMP) production which in turn reduce protein kinase G (PKG) activity in cardiomyocytes and consequently titin protein function and diastolic distensibility (21, 34). Similarly, AGEs impair endothelial NO production and predisposes to concentric LV remodeling and myocardial stiffness as observed in diabetic cardiomyopathy patients with HFpEF (35–38). In addition, in T2DM there is an increase in glucose-auto-oxidation and free-fatty acid concentrations which creates oxidative stress in the myocardium and subsequently concentric ventricular remodeling (39). Lam (30) recognized T2DM-related HFpEF as a unique diabetic cardiomyopathy phenotype, which can be defined by the presence of left ventricular diastolic dysfunction in diabetic patients without coronary artery disease, hypertension, or other potential etiologies (40). This phenotype is in contrast with the well-established diabetic cardiomyopathy definitions where LV dilatation, reduced EF and systolic dysfunction are the main characteristics (30). The features of both phenotypes have been compared in detail elsewhere (21). Common HFpEF mechanisms associated with obesity and T2DM are summarized in Table 1. Like obesity, T2DM patients may develop EAT expansion, which contributed to HFpEF pathogenesis *via* several mechanisms.

**Table 1:** Common obesity and type 2 diabetes mellitus related HFpEF mechanisms.

- Systemic inflammation - Expansion of epicardial adipose tissue (EAT) - “Cardiac steatosis” - Cardiac fibrosis - Increased endothelial production of reactive oxygen species - Impaired endothelial nitric oxide production - Increased myocyte stiffness

## The Role of Epicardial Fat in Obese or Diabetic HFpEF Patients

**Figure 1:**
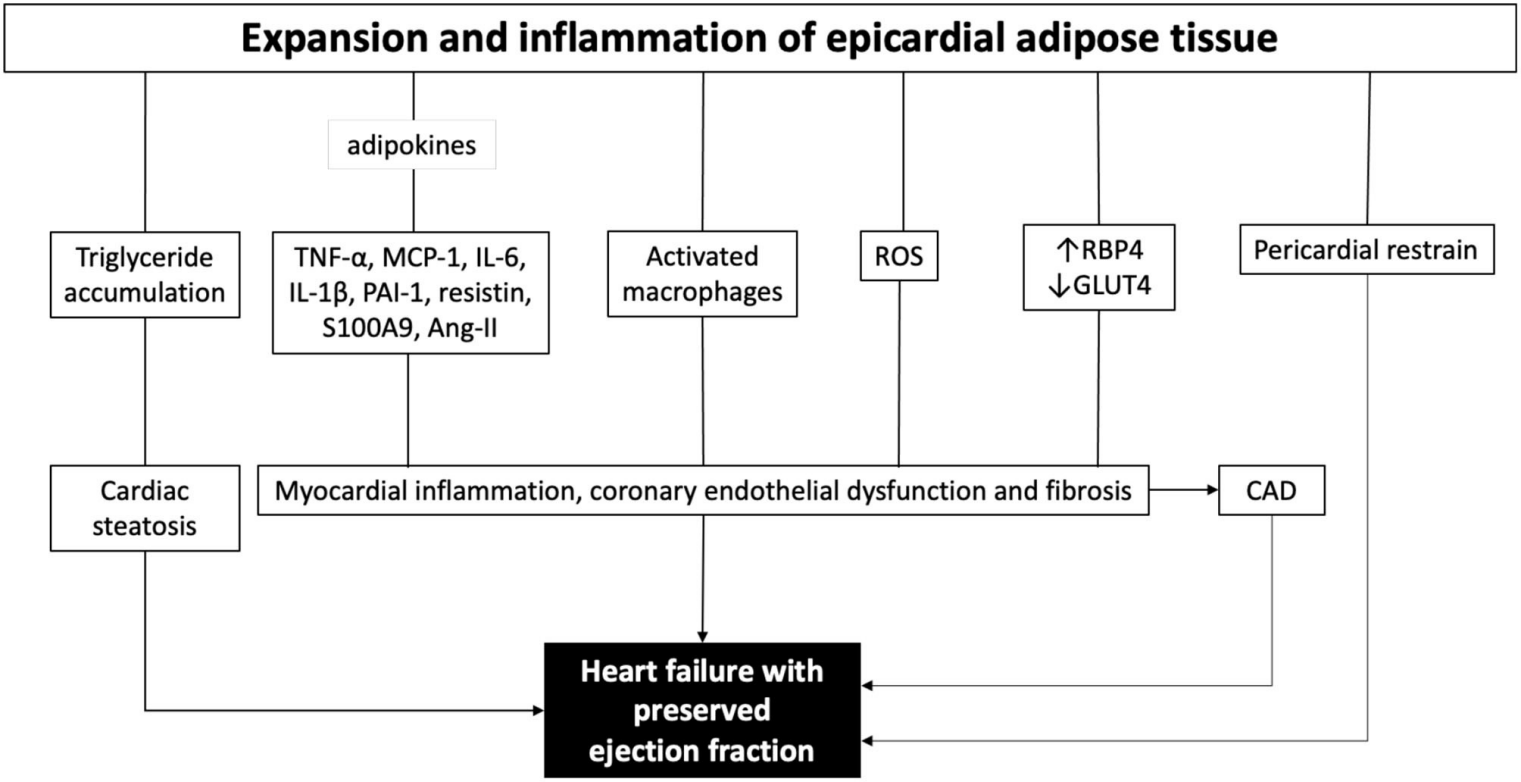
The role of epicardial adipose tissue in the pathogenesis of heart failure with preserved ejection fraction. ROS, reactive oxygen species; TNF-α, tumor necrosis factor alpha; MCP-1, monocyte chemoattractant protein 1; IL-6, interleukin 6; IL-1b, interleukin 1b; PAI-1, plasminogen activator inhibitor-1; Ang-II, angiotensin II; ROS, reactive oxygen species; RBP4, renitol-binding protein 4; GLUT4, glucose transporter-4; CAD, coronary artery disease.

EAT is the visceral fat depot of the heart. In the adult, EAT is physiologically found in the atrioventricular and interventricular grooves of the heart. HFpEF patients have 20–50% higher EAT mass compared to both patients with non-obese HFpEF and control subjects with similar body mass index (14–16). EAT expansion includes the intensification of perivascular fat which causes coronary inflammation and accelerated atherosclerosis, and ultimately leads to myocardial stiffness and fibrosis (41, 42). The EAT interacts directly with the heart, metabolically and mechanically (Figure 1) (15, 17). Since there is no muscle fascia between the EAT and the myocardium, the two tissues depend on the same microvasculature and could interact directly *via* paracrine and vasocrine secretions (43, 44). In obese patients, the EAT secretes several pro-inflammatory chemokines and cytokines, collectively called adipokines such as tumor necrosis factor alpha (TNF-α), monocyte chemoattractant protein-1 (MCP-1), interleukin-6 (IL-6), IL-1β, plasminogen activator inhibitor-1 (PAI-1), resistin, S100A9, and many others (16). All together create a proinflammatory state in the myocardium associated with cardiomyocyte stiffness, coronary endothelial dysfunction, and fibrosis, which are all implicated in the development of HFpEF (14, 15, 45). In line, Karastergiou et al. (46) have reported a strong presence of activated macrophages in the EAT obtained from obese patients with coronary artery disease (CAD) (46). Moreover, the EAT produces high levels of reactive oxygen species (ROS) products, which drives oxidative stress in the myocardium and the coronary vasculature (47). Furthermore, the EAT is a source of angiotensin II, which provokes coronary vasoconstriction enhancing ischemia, especially in patients with DM due to coexisting vasculopathies (48). Also, the EAT transcriptome contributes to the development of CAD *via* higher levels of renitol-binding protein 4 (RBP4) and lower levels of glucose transporter-4 (GLUT4), leading to adverse lipid and glucose metabolic profile augmented by the proinflammatory secretome (49, 50). Metabolically, EAT expansion is associated with intramyocardial accumulation of triglycerides causing cardiac steatosis (51). It has been shown that myocardial triglyceride content is independently associated with reduced pump function (52), and impaired ventricular strain parameters (53). Cardiac steatosis induces fetal gene transcription that favors myocardial glucose utilization instead of free fatty acids under physiological conditions, which further aggravates lipid accumulation (54, 55).

Mechanically, the EAT occupies space in the cardiac fossa. In obese patients, large EAT mass causes an increase in intracardiac pressures, particularly during exercise (15, 56). In HFpEF patients, an expansion in the EAT volume is commonly observed jointly with biventricular hypertrophy, however, the pericardium does not expand at the same proportion (15, 56). Hence, the pericardium exerts a compressive contact force on the heart and consequently increases pericardial restrain and enhances ventricular interaction (57). The abnormal mechanical interaction between the heart and the pericardium causes an increase in pericardial pressure and LV end-diastolic pressure, and a decrease in LV transmural pressure and LV end-diastolic diameter which together elevate the pulmonary capillary hydrostatic pressures promoting dyspnea (58). Further studies have substantiated the deleterious effect of EAT expansion on the cardiac muscle function including strain abnormalities (59). In a 2-dimensional speckle tracking echocardiography study, Maimaituxun et al. (60) have demonstrated that EAT volume is a determinant of global longitudinal strain (GLS) in HFpEF patients. Similarly, DM was a sole determinant of GLS (60).

Visceral fat accumulation is a fundamental element of T2DM (43, 61). HFpEF patients with T2DM have higher EAT mass compared to those without T2DM at similar BMI (16, 61). Being a marker of visceral adiposity, EAT is a risk factor for T2DM, cardiovascular complications and metabolic syndrome (14, 44, 62, 63). In diabetics, the EAT transcriptome is rich in proinflammatory and innate immune genes like Pentraxin 3 (PTX3) and endothelial lipase G (LIPG) compared with the subcutaneous fat transcriptome from the same patients (64). Van Woerden et al. (16) have observed that in HF patients creatine kinase-MB, troponin T, and glycated hemoglobin positively correlate with EAT volume. It appears that EAT expansion is associated with DM, however, whether there is a causal association between the two disorders is not yet clear.

## Diagnosis of Epicardial Adipose Tissue Expansion

The diagnosis of EAT enlargement is imperative for the identification of EAT-related HFpEF phenotypes. Transthoracic echocardiography can estimate the EAT thickness, measured as the echo-lucent area between the epicardial surface and parietal pericardium. However, echocardiography cannot be used to estimate EAT volume and has relatively poor inter-observer and intra-observer variability among other limitations (65).

**Figure 2:**
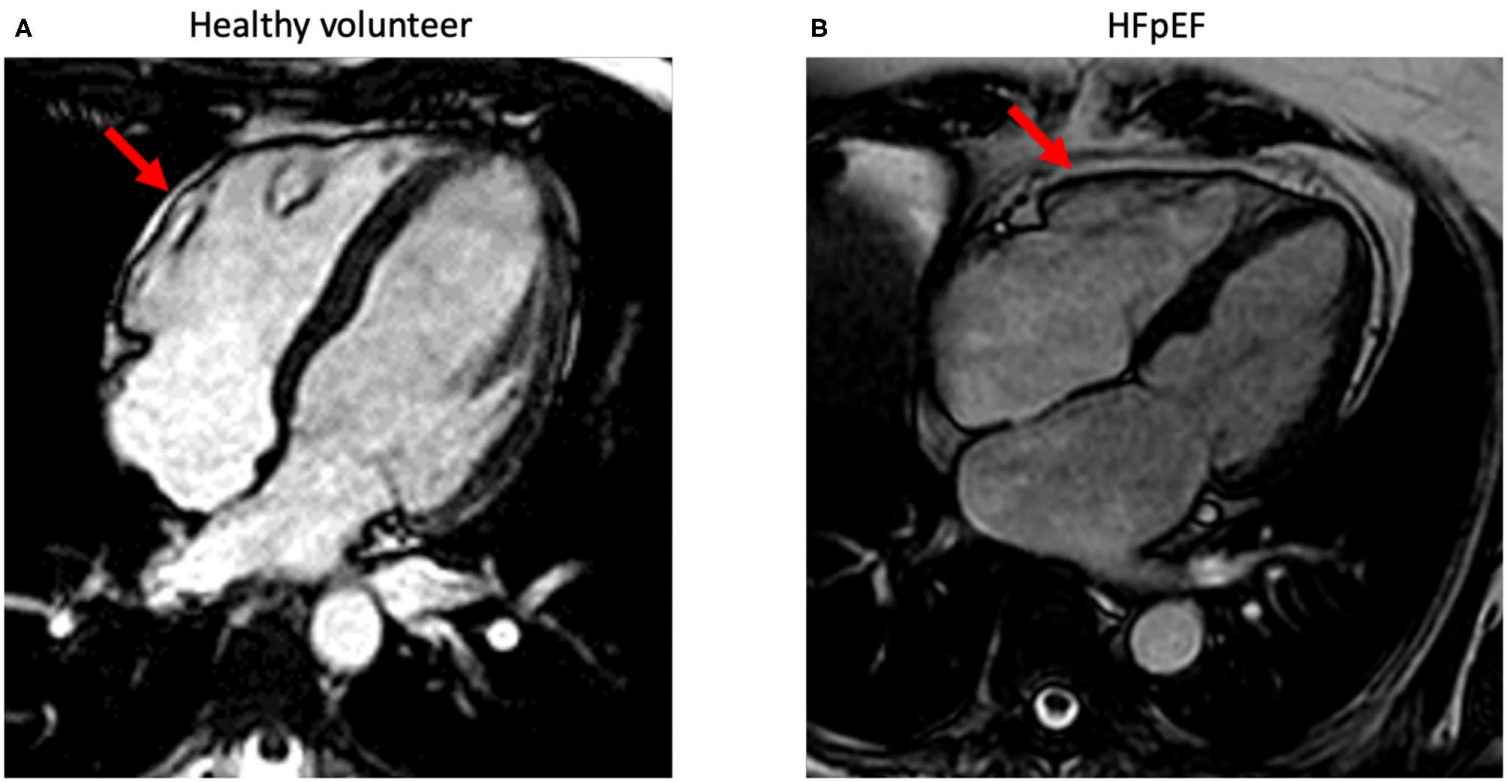
Standard-4-chamber orientations acquired using standard steady-state free precession-cardiac magnetic imaging sequences. The images demonstrate epicardial fat (red arrow) with a minimal (normal) amount in a healthy volunteer **(A)** and in a patient with HFpEF **(B)** with increased epicardial fat surrounding the whole heart.

Ideally, EAT volume can be evaluated *via* cardiac magnetic resonance (CMR) (66, 67) (Figure 2). In line, the European society of cardiology (ESC) consensus recommends the use of a stepwise score-based algorithm to diagnose HFpEF (68). The algorithm suggests the use of more sophisticated tools including CMR to identify specific etiologies in patients with confirmed HFpEF. CMR-based stratification of HFpEF patients based on EAT volume might support the rational to use of EAT-modulating therapies. In addition, CMR opens the possibility to a combined evaluation of potential underlying myocardial ischemia, storage diseases like amyloidosis, diffuse myocardial fibrosis (extracellular volume), and epicardial fat. Recently, a growing number of publications also demonstrate the easiness of epicardial fat quantification using artificial intelligence (AI) algorithms (69). Beside the heart, the same approach can also be applied to other organ regions in patients with HFpEF like the abdomen (70). Since CMR allows imaging also in severely obese patients, functional evaluation of the systolic and diastolic function including myocardial work is feasible (71). Recent guidelines of the ESC recommend the use of the imaging modality which is locally available with the best expertise and confidence in results. Cardiac computed tomography (CT) is widely available and easy to use and has demonstrated its ability to quantify epicardial fat. However, the necessary use of radiation limits its application for serial measurements needed to control treatment effects. However, since newest generation CT-scanner can be used with very low radiation doses, this might be an interesting field for future research. In addition, quantification of EAT by cardiac CT can be used to predict outcome even in inflammatory diseases like Covid-19 (72).

## Therapeutic Strategies

### Lifestyle Modification

Unlike HF with reduced EF (HFrEF), patients with HFpEF phenotype do not benefit from most standard HF therapies, but rather from lifestyle modifications (73, 74). Similarly, diabetic cardiomyopathy patients with an HFpEF phenotype benefit more from lifestyle modifications compared to standard heart failure therapies (21). Implementation of a healthy lifestyle including smoking cessation, weight reduction, exercise, and healthy diet is the mainstay of HFpEF management (75). Looking at obese-HFpEF patients, weight reduction, and exercise might regress the EAT volume, restore its physiological role (14), and mitigate excessive pericardial restraint (15).

### Symptomatic Treatment With Diuretics

In addition to lifestyle modifications, the ESC 2016 guidelines have recommended the symptomatic use of diuretics in HFpEF (3). Owing to the strong dependency between filling pressures and blood volume, obese-HFpEF patients might benefit from diuretics (15, 76). By reducing blood volume, diuretics reduce right ventricular volume and ventricular interaction, which in turn improves LV end-diastolic volume (LVEDV) and stroke volume (SV), and relives pericardial restrain (15, 77). However, the diuretic dose should be carefully adjusted to avoid hypovolemia and severe preload reduction (3).

### Epicardial Fat Modifying Interventions

#### Statins and Other Antihyperlipidemics

Statins are lead prescribed medications that can achieve substantial serum cholesterol lowering *via* inhibiting 3-hydroxy-3-methylglutaryl coenzyme A (HMG-CoA) reductase, the rate-limiting enzyme of cholesterol synthesis (78). Members of this class are known pleiotropic agents having the potential to affect different body tissues independent of lipid lowering (79, 80). Statins restore endothelial redox balance and NO bioavailability (81). Although the use of statins in HFpEF has been a topic of debate, several studies support the beneficial effect of statins in HFpEF (82, 83). Various metanalysis studies advocate the potential benefit of statins on HFpEF-mortality (84–86). In cohort studies statins have demonstrated the ability to reduce EAT volume up to 15% with atorvastatin, independent of low-density lipoprotein cholesterol-lowering (79, 87). Prominently, a multi-center study comparing 87 aortic stenosis patients on statins to 106 not on statins, concluded that statin treatment was significantly associated with reduced EAT thickness and proinflammatory cytokines secretome (88). The expression of LDL and VLDL receptors by the EAT, advocates the role of statins in modulating the metabolism of this fat depot (89). Besides, it has been suggested that statins reduce the EAT metabolic activity (79). Ultimately, the exact mechanism by which statins affect the EAT volume is so far unknown.

Ezetimibe is another antihypercholesterolemic agent that inhibits cholesterol absorption from the gut. In combination with simvastatin, ezetimibe could not achieve better reduction in EAT thickness compared to atorvastatin monotherapy (87).

Proprotein convertase subtilisin/kexin type 9 inhibitors (PCSK9i) are novel cholesterol lowering agents which act *via* inhibiting the PCSK9 enzyme which is responsible the downregulation of low-density lipoprotein (LDL) receptors on the surface of hepatocytes (90). A study by Galvez et al. concluded that 6 months of treatment with PSCK9i could achieve a 20% reduction in EAT thickness (91).

#### Metformin

Metformin is the first-line treatment for T2DM, particularly in obese patients. Alongside its glucose-lowering effect, metformin use is associated with weight loss independent of diabetes, primary due to reduction of VAT (92, 93). A recent meta-analysis by Halabi et al. concluded that metformin treatment is associated with a reduction in mortality in patients with HFpEF (94). In addition, two recent studies have demonstrated that metformin monotherapy reduces EAT thickness (95, 96). A standard metformin monotherapy for 3 months could reduce the EAT thickness by 10% (95). The exact mechanism by which metformin affects EAT is unclear but most reasonably attributed to the well-established metabolic effects of metformin, specifically shifting metabolism into fat oxidation and upregulation of thermogenesis (92, 95).

#### Glucagon-Like Peptide-1 Receptor Agonists

Semaglutide, liraglutide, and dulaglutide are glucagon-like peptide-1 (GLP-1) receptor agonists, indicated for the treatment T2DM. They act *via* enhancing glucose-dependent insulin secretion, reducing glucagon secretion, and delaying gastric emptying, resulting in adequate T2DM control and weight loss (97). Several studies have demonstrated that the use of GLP-1 associates with reduced cardiovascular risk (98, 99). In a randomized placebo-controlled trial on patients with T2DM, liraglutide exhibited favorable cardiovascular outcomes including LV filling pressure reduction and diastolic function improvement. Both parameters are relevant for diabetic cardiomyopathy and HFpEF (100). In addition, a systematic review comparing the effect of GLP-1 agonists to different antidiabetics on LV diastolic function has concluded that liraglutide monotherapy offers a considerably beneficial outcome (101). Interestingly, the EAT was found to express GLP-1 receptors in contrast to subcutaneous fat in the same patient (102). In a cohort of patients with T2DM and obesity, Lacobellis and his colleagues demonstrated that weekly administration of either semaglutide or dulaglutide causes rapid, substantial, and dose dependent reduction in EAT thickness, attaining 20% reduction in 12 weeks (103). Similarly, liraglutide treatment on top of metformin has resulted in 29 and 36% reduction in EAT after 3 and 6 months, respectively, together with a reduction in BMI and glycated hemoglobin (104). GLP-1 agonists are suggested to regulate EAT adipocyte formation and metabolism as an outcome of promoting preadipocyte differentiation, increasing sensitivity to insulin and stimulating thermogenesis and adipocyte browning *via* a complex signaling system (105–107).

#### Dipeptidyl Peptidase-4 Inhibitors

Endogenous GLP-1 is susceptible to cleavage by dipeptidyl peptidase-4 (DPP-4). Sitagliptin, a DPP-4 inhibitor, has recently been shown to substantially reduce EAT in subjects with obesity and T2DM *via* prolonging GLP-1 half-life (108). DDP-4 inhibitors are recommended in T2DM patients without cardiovascular risk. Whether DPP-4 inhibitors would offer long-term benefit for HFpEF patients or not is debatable. Several studies have shown that DPP-4 inhibitors exhibit cardioprotective anti-inflammatory properties that may have beneficial effects on EAT (109–111). Those effects were described to be mediated *via* different mechanisms including downregulation of the receptor for AGE (RAGE) (112), activation of cAMP/PKA signaling and IL-6 production (113), reduction of ROS generation and ICAM-1 expression (114), and diminishing DPP-4-activated phosphatidylinositol 3-kinase signaling which favors adipocyte maturation (115). In contrast, other studies have concluded that DPP-4 inhibitors might increase the inflammatory products of the EAT and adversely affect the myocardium especially in diabetic patients *via* potentiating the actions of endogenous proinflammatory chemokines like CXCL12 and mineralocorticoid receptor signaling (116–118).

#### Sodium-Glucose Cotransporter 2 Inhibitors

Sodium-glucose cotransporter 2 inhibitors (SGLT2i), for example dapagliflozin and empagliflozin are relatively new medications indicated for T2DM patients, but they are also sufficient in HFrEF patients without DM (119, 120). Agents of this class lower plasma glucose concentration *via* increasing urinary glucose excretion (121). In addition, they cause significant weight reduction, comparable to GLP-1 agonists, *via* caloric loss, osmotic diuresis, and stimulation of visceral fat burn (122, 123). Like diuretics, SGLT2i can reduce plasma volume and consequently ventricular filling pressures, offering benefit to HFpEF patients (15). With respect to HFpEF animal data, showing that SGLT2 inhibition exhibited beneficial cardiovascular effects in several non-diabetic HFpEF-animal models (124, 125). Cardiomyocytes from empagliflozin-treated HFpEF patients showed improved NO–sGC–cGMP–cascade and PKG-activity, suggesting favorable cardiovascular outcomes (126). In patients with T2DM and recent worsening HF, sotagliflozin treatment reduced the total number of deaths from cardiovascular causes and hospitalizations and urgent visits for HF, particularly in patients with preserved EF (127). Press release about the EMPEROR-Preserved study, which has been recently completed on HFpEF patients, stated that the study met its primary endpoint showing that empagliflozin has reduced mortality and hospitalization in the treated cohort [NCT03057951]. Moreover, optimistic results are expected from the DELIVER trial, which investigates the benefit of dapagliflozin in HFpEF patients (128), all showing that SGLTi inhibition can exert cardio-beneficial effects in HFpEF. In addition, in T2DM patients dapagliflozin could reduce the EAT volume and the occurrence cardiovascular events (94). Similarly, dapagliflozin was recently shown to cause significant EAT thickness reduction reaching 20% after 24 weeks of treatment in obese patients with T2DM, independent of weight loss (129). Weight loss independent mechanisms include improvement of EAT cells-insulin sensitivity and reduction of local proinflammatory chemokines secretion (e.g., CCL2) (130). Overall, SGLT2i showed favorable results in HFpEF patients including EAT reduction. Whether this effect is mediated *via* weight loss, or a direct metabolic mechanism warrants further investigation.

### Surgical Pericardiectomy

Anterior pericardiotomy through minimally invasive percutaneous procedure is a potentially novel last-option treatment for HFpEF patients with severe LV restriction, since it eliminates the external restraint of the pericardium (57, 131, 132). In canine HFpEF animal models, resection of the pericardium improved LV compliance and filling pressure (132). This technique is also applicable in humans, however further studies are warranted to evaluate its long-term benefit and safety (131).

### Future Therapies

#### Anti-inflammatory Agents

Inflammation is an important driver of HF, by which its role in the pathogenesis of HFrEF and HFpEF differs (133, 134). Although it plays a pathological role in the EAT of obese patients (46), pharmacological treatment with anti-inflammatory agents like steroids (135) cannot be recommended in HF patients with DM and/or metabolic syndrome (136). Theoretically, several biological agents like interleukin (IL)-1 and IL-6 inhibitors can target EAT-induced myocardial inflammation (136). The small D-HARD study has demonstrated the beneficial effects of the competitive IL-1 receptor antagonist anakinra in HFpEF patients (137). However, the subsequent phase II (D-HART2) study has failed to corroborate favorable outcomes (138). Eventually, the study was underpowered and most of the study participants suffered from obesity which independently affects the co-primary endpoints peak oxygen uptake (V_O2_) and ventilatory efficiency (V_E_/V_CO2_) (138). Whether the D-HART2 study results be different in HFpEF patients with enlarged EAT warrants further investigation. Canakinumab is another monoclonal antibody that binds and neutralizes IL-1β. Results from the CANTOS study demonstrated that canakinumab significantly reduces the recurrence of new cardiovascular events (139). These findings form the rationale to consider investigating whether canakinumab would influence EAT-inflammation. Ultimately, clinical studies testing whether targeting EAT-related proinflammatory cytokines can benefit HFpEF patients with enlarged EAT or not are necessary.

## Conclusion

HFpEF is a diverse disease resulting from wide range of comorbidities. Obesity and DM are principal drivers of HFpEF. Stratifying HFpEF patients based on phenotypic data results in novel classifications including obese and diabetic HFpEF phenotypes. There is a close association between EAT volume and HFpEF. HFpEF patients can be further classified according to EAT volume using advanced imaging techniques including CMR and CT. EAT functions as endocrine tissue that contributes to myocardial inflammation. In addition, EAT expansion acts as space-occupying lesion that causes pericardial restrain, increase in ventricular filling pressures, and enhanced ventricular interaction. HFpEF patients with enlarged EAT may benefit from lifestyle modifications and symptomatic treatment with diuretics. Besides, statins, PCSK9i and fat-modulating anti-diabetic agents like metformin, SGLT2i or GLP-1 agonists can be especially effective in this subgroup of patients, being able to induce EAT regression. In addition, direct effects of SGLT2i and GLP-1 agonists on HFpEF are currently under clinical investigation.

## Author Contributions

AE wrote most of the manuscript and prepared the figures. VN wrote short parts of the manuscript. SV revised the manuscript and applied changes to the text and figures. CT provided the main ideas of the manuscript and guided the other authors, revised the manuscript, and added some parts. SK provided Figure 2 and wrote short parts of the manuscript. All authors contributed to the article and approved the submitted version.

## Conflict of Interest

CT has received speaker fees and/or contributions to congresses from Abbott, Abiomed, Astra Zeneca, Bayer, Böhringer-Ingelheim, Novartis Pfizer, and Servier; all outside the submitted work. The remaining authors declare that the research was conducted in the absence of any commercial or financial relationships that could be construed as a potential conflict of interest.

## Publisher's Note

All claims expressed in this article are solely those of the authors and do not necessarily represent those of their affiliated organizations, or those of the publisher, the editors and the reviewers. Any product that may be evaluated in this article, or claim that may be made by its manufacturer, is not guaranteed or endorsed by the publisher.
